# Anti-infective Surface Coatings: Design and Therapeutic Promise against Device-Associated Infections

**DOI:** 10.1371/journal.ppat.1005598

**Published:** 2016-06-02

**Authors:** Bryan R. Coad, Hans J. Griesser, Anton Y. Peleg, Ana Traven

**Affiliations:** 1 Future Industries Institute, University of South Australia, Mawson Lakes, South Australia, Australia; 2 Infection and Immunity Program and the Department of Microbiology, Biomedicine Discovery Institute, Monash University, Clayton, Victoria, Australia; 3 Department of Infectious Diseases, Central Clinical School, Alfred Hospital and Monash University, Melbourne, Victoria, Australia; 4 Infection and Immunity Program and the Department of Biochemistry and Molecular Biology, Biomedicine Discovery Institute, Monash University, Clayton, Victoria, Australia; Geisel School of Medicine at Dartmouth, UNITED STATES

## Introduction

Patient safety and well-being are under increasing threat from hospital-acquired infections [1]. The root cause of a large number of these infections arises from microbial biofilms that colonise on surfaces of medical devices such as the millions of catheters, endotracheal tubes, and prosthetics implanted every year [2]. Biofilm infections are accompanied by increased resistance to antimicrobial therapy and immune clearance, severely limiting treatment options and leading to life-threatening disease [3,4]. Device-associated infections are caused by both bacteria and fungi and, while most studies have focused on single-species biofilms, biofilm-related infections are often polymicrobial [5–8]. Multi-species biofilms, particularly those involving bacterial and fungal pathogens, are more challenging to treat, likely as a consequence of their combined architecture, protective extracellular matrix, and potential synergism in protecting against antimicrobials and host immunity [9–11]. Among the fungi, *Candida* species are the most important biofilm pathogens [12,13] and the fourth leading cause of blood-stream infections in United States hospitals [7]. Fungal diseases remain difficult to diagnose, mortality rates remain high, and antifungal drug resistance continues to limit therapeutic options [14,15]. We are in desperate need of innovative strategies that target the mechanisms of pathogenesis of polymicrobial biofilms on medical devices. This is a grand challenge because it requires multi-disciplinary collaboration and breakthrough research involving physical chemistry, materials science, and microbiology. Communication between these disciplines has not been common, but recent advances show greater convergence in the development of anti-infective devices. At this nexus, we outline the therapeutic promise of anti-infective coatings for medical devices and discuss pitfalls and strategies for overcoming them.

## Stopping Biofilm Formation before It Starts

A key step in biofilm formation is the initial adherence to the surface [16,17]. Once attached, the infectious agents replicate, colonise the device, and embed themselves within a protective extracellular matrix. Biofilm drug resistance is thought to be caused by several mechanisms, including sequestering of drugs, persister cells, and specific aspects of biofilm cell physiology [18,19]. One solution is to kill or prevent adhesion of pathogens on medical devices via delivery of antibiotics from the surface of materials/devices, which would allow for a high concentration of the drugs at the implantation site [20]. Research has been devoted to novel antimicrobial agents such as cationic compounds, semi or fully synthetic antimicrobial peptides, or the use of silver [21–24]. While these approaches have significant advantages, such as the ability to physically disrupt the microbial membrane (a mechanism by which microbes may have difficulty developing resistance), there are also disadvantages. One is that the safety of such compounds needs to be demonstrated to show selectivity for preferentially lysing only pathogenic membranes over mammalian cells—and importantly, this window of selectivity becomes even narrower when discriminating between eukaryotic cell types (e.g., fungi and mammalian cells). As a result, new compounds will require a prolonged regulatory approval process to demonstrate their safety, ultimately slowing development. Devices utilising commercially available pharmaceuticals would expedite the development process. In either case, particularly for vascular devices, the potential for interaction between surface compounds and drugs or other compounds administered via the device will require testing to ensure regulatory standard. In light of these issues, we focus this short review towards antimicrobial surfaces that use approved pharmaceuticals.

**Fig 1:**
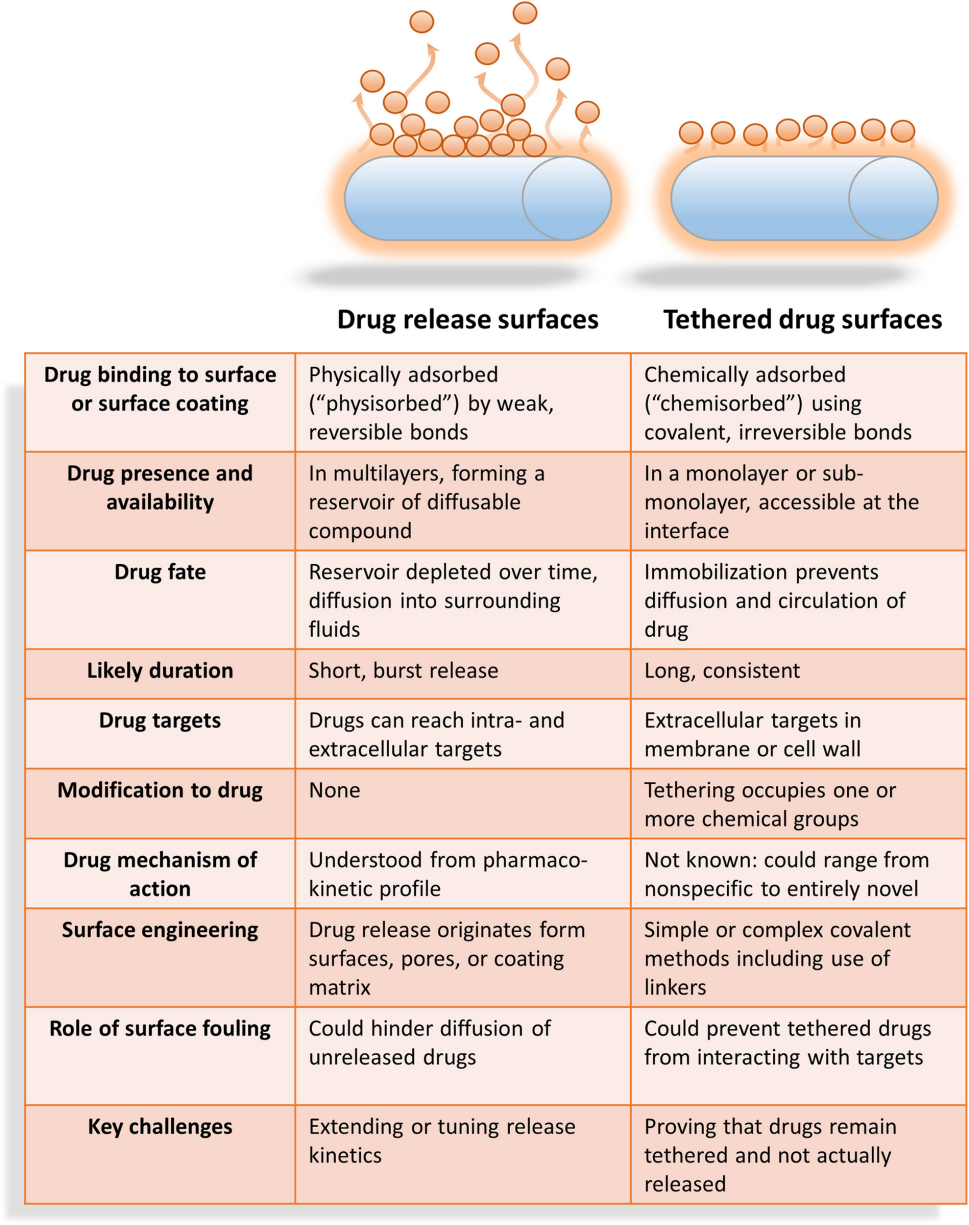
Important factors in the design of anti-infective materials and their surface coatings.

One way to deliver antimicrobial pharmaceuticals is by coatings that act by “drug release.” This approach involves pre-loading the surface coating with a reservoir of drug that, once implanted, gradually diffuses into the fluids and tissues surrounding the implant, providing a high local dose of the drug [25]. Another strategy is “tethered drug surfaces,” on which the antimicrobial pharmaceuticals are attached by means of chemical bonds directly to the surface or a surface interlayer. Fig 1 compares the key aspects of these coatings and lists advantages and disadvantages relating to their possible clinical use and mechanism of action.

Whether novel or approved antimicrobial agents are used, the ability for infectious agents to develop resistance is a growing concern. Drug-releasing coatings create a local concentration gradient, reaching diminishingly small concentrations (eventually below the Minimum Inhibitory Concentration [MIC]) that may promote antimicrobial resistance in the context of a localised infection. Tethered coatings maintain a high local concentration on the device periphery, which circumvents this issue [26].

## Surface-Attached Pharmaceuticals: Promises

Tethered drug surfaces are a particularly interesting area of fundamental research because of the potential to uncover new mechanisms of action for “old” drugs. In other words, we are greatly interested in how the antimicrobials are presented from the surface, whether they would find existing or new targets within the cell wall or membrane, and the chemical and biophysical result of interactions with microbes that might delay the acquisition of resistance phenotypes. These mechanisms could be different from their solution-based understanding, thus driving innovation in drug formulation [27]. This particular aspect would be of great commercial interest to pharmaceutical companies or drug formulators as an opportunity to add value to, or find novel mechanisms for, their existing or non-profitable chemical libraries.

## Surface-Attached Pharmaceuticals: Pitfalls

Tethered drug surfaces are prepared by forming a chemical bond between functional groups on the drug target and groups on the surface or surface coating. In order for the benefits of a tethered drug surface to be achieved (Fig 1 and see below for additional discussion) it is essential that (1) the drug is and remains covalently bound to the surface and (2) it can be analytically verified that drugs are not released from the surface. The first point presents a major technical challenge because large excesses of compounds must be used when preparing surfaces [28]. When excess compound is present during binding, only a small fraction of drug can become chemically attached in a monolayer (i.e., chemisorbed or irreversibly bound through the formation of a covalent bond) to the surface. The major portion will instead much more readily associate weakly into physically adsorbed (i.e., physisorbed) layers or multilayers, in which drugs are held together by weak interfacial bonds that have the potential to be reversed over time or in different solution environments (e.g., ionic strength, pH, polarity, and physiological temperature). While washing steps are used to remove this excess, there is a pitfall in assuming that one (or even multiple) washing steps will be sufficient because washing solutions might not be able to readily disrupt weak bonds.

For antimicrobial pharmaceuticals that inhibit cell wall synthesis or otherwise compromise cell wall integrity, tethering them to surfaces is a logical strategy, thus allowing them to interface with targets in the cell envelope. One promising candidate is caspofungin: an antifungal lipopeptide that inhibits 1, 3 β glucan synthase and thus disrupts fungal cell wall biogenesis [29]. Another is vancomycin, which binds to cell wall peptides of Gram-positive bacteria and prevents crosslinking [30]. Coad et al. demonstrated that a covalent caspofungin coating that can be applied to a variety of biomaterials kills the four most common *Candida* species causing device-associated infections [31]. Kucharíková et al. demonstrated both caspofungin and vancomycin tethering to surface coatings (separately) on titanium implant devices and showed their in vitro and in vivo efficacy against *Candida albicans* and *Staphylococcus aureus* [32]. To understand the mechanism of action, analytical evidence can demonstrate whether these antimicrobial agents are covalently attached to their surface or whether they might reversibly desorb. Surface-sensitive techniques such as X-ray photoelectron spectroscopy (XPS) and time-of-flight secondary ion mass spectrometry (ToF-SIMS) can be used to show the presence of caspofungin on surface coatings. Using these analytical approaches, it has been demonstrated that even after extensive water and buffer washing, physisorption dominated over covalent attachment and was responsible for the majority of the caspofungin detected (66%) [31]. Only after extended washing with elevated temperature and surfactant could all traces of physisorbed compound be removed. These covalently bound caspofungin samples were effective using in vitro studies [31]. Other analytical techniques such as high performance liquid chromatography (HPLC) and scanning electron microscopy (SEM) have also been used to provide quantitative and qualitative evidence for the presence of bound drugs. Using these techniques, vancomycin was shown to bind to surfaces with monolayer coverage, which is possible through physisorption and/or chemisorption [32]. The same study found that caspofungin binding resulted in multilayers [32], which is only possible when physisorbed, thus suggesting that only a fraction of the compound was covalently attached to the surface.

Further studies with tethered drugs illustrate the importance of assessing the physical or chemical properties of surfaces and their coatings. Antibacterial drugs such as tetracycline and levofloxacin require penetration of the drug into the cell cytosol to interact with their intercellular targets. Therefore, surface coatings with these drugs tethered would need to provide sufficient linker length and conformational flexibility to penetrate through the network of membrane and cell wall biomolecules to span the cell envelope thickness of approximately 40–50 nm [33]. With tetracycline, Davidson et al. suggested that covalent attachment of the drug to ethylene oxide linkers was responsible for penetration into the Gram-negative cell wall allowing for elimination of *Escherichia coli* [34]. The linkers used in the chemical synthesis could theoretically stretch to 6–7 nm in length, but the physical forces governing chain conformations in biological fluids would further limit the distance in vivo. Thus an alternative explanation for antibacterial effect is likely through a novel mechanism of action or release of the drug from the surface entering the cell. Consistent with the idea that release of adsorbed compounds by desorption or hydrolysis could explain the mode of action of intercellularly acting drugs, Kugel covalently attached levofloxacin to surfaces and observed killing of *E*. *coli*, and the antibacterial effect was suggested to be release of levofloxacin from hydrolytically unstable tethers [35].

Compared to antibacterial coatings, the need for developing antifungal biomaterials has received far less attention [27], but it is an essential prerequisite for developing hybrid strategies for combatting polymicrobial device infections. As illustrated by the examples above [31,32], the demonstrated antifungal effect in vitro and in vivo of surfaces coated with caspofungin show promise for future development of device coatings that could be beneficial against polymicrobial infections. The need to design materials that eliminate multi-species or cross-domain infections will require hybrid approaches that could involve not only pharmaceuticals but also novel agents such as cationic compounds or antimicrobial peptides that are broadly antibacterial and antifungal and likely to incorporate both tethering and releasing strategies. However, caution will be needed to ensure that these coatings are safe and compatible with host cells.

## Complex Problems Requiring Multidisciplinary Solutions

Advances in coatings for biomedical devices are likely to develop from new, fundamental studies in materials science, engineering, and nanotechnology. It can be seen that in these disciplines, an increasing number of publications focus on antimicrobial surface coatings and their understanding [27]. As discussed, there are challenges in fabricating anti-infective tethered drug coatings, but recent work has shown that covalent attachment of antimicrobial compounds to surfaces is achievable and effective, although more understanding is needed. Surfaces with non-desorbing compounds that operate through novel, surface-associated mechanisms of action will be an innovative component of future anti-infective medical device surface coatings that are long lasting. As described above, the important role of physisorption, hydrolysis, and chemical structure should be considered in evaluating the mechanism of action. Progress will also be helped by knowledge about surface-sensitive analytical instrumentation, the strengths and limitations of such techniques, and their role in understanding surface phenomena. Admittedly, the need for better physicochemical understanding of surfaces by materials scientists is not being communicated to the wider biological community, likely due to lack of discourse between disciplines. So, too, is a greater need for materials-led research to be relevant to the biological and clinical communities so that it becomes an effective tool for the microbial pathogenesis community, clinician, and end-user. Multidisciplinary consortia to tackle these important challenges and educating peers to critically evaluate this work will pave the path to developing effective and novel therapeutic strategies for problematic biofilm-related infections.
